# Provenance and Clinical Benefit of Medicines Introduced to the French Market, 2008 to 2018

**DOI:** 10.1001/jamainternmed.2023.6249

**Published:** 2023-11-20

**Authors:** Leeza Osipenko, Philippe Potey, Bernardo Perez, Alexandra Kupryjanczuk, Filip Angelov, Alexandra Schuster, Elias Mossialos

**Affiliations:** 1 Department of Health Policy, London School of Economics, London, United Kingdom; 2 Consilium Scientific, London, United Kingdom; 3 Queen’s Medical Research Institute, University of Edinburgh, United Kingdom; 4 Department of Innovation, Cleveland Clinic Florida, Weston; 5 Department of Health Technology, Technical University of Denmark, Kongens Lyngby, Denmark

## Abstract

**Importance:**

Both the commercial sector and academia play a vital role in medicine development. Ongoing debates exist on their contribution and the value of medicinal products entering the market.

**Objective:**

To identify the provenance and clinical benefit of medicines that entered the French market between 2008 and 2018.

**Design and Setting:**

In this cross-sectional study, the provenance of each medicine in the French market was established via a review of multiple sources documenting at least 2 matching findings per product. The clinical benefit was assigned using the matched scale developed from the Prescrire and Haute Autorité de Santé (HAS) gradings. The χ^2^ test was used to analyze the proportions and frequencies of medicines graded by Prescrire and HAS by origin, therapeutic category, and clinical benefit.

**Main outcomes and measures:**

The origins and therapeutic categories of medicines. Clinical benefit based on Prescrire and HAS grading. Concordance of Prescrire and HAS grading.

**Results:**

Of the 632 medicines that entered the French market between 2008 and 2018, 464 originated (73%) in the commercial sector, and 168 originated (27%) in the academic setting or in collaboration with commercial enterprises. Prescrire graded psychotropic agents (13/14 [93%]), whereas HAS graded respiratory agents (24/25 [96%]) as the highest percentage of medicines that provided no added benefit. Prescrire graded 360 medicines (77.6%) that originated in the industry and 108 medicines (64.3%) that originated in the academic setting (*P* = .001) to have no added clinical benefit. HAS assigned such grading to 331 ([71.3%] industry) vs 104 ([61.9%] academia) (*P* = .02). Based on the Prescrire grading, academia invented more medicines delivering some added benefit 57 (33.9%) vs 98 (21.1%) invented by industry (*P* = .001). HAS grading on some added benefit 51 ([30.4%] academia) vs 121 ([26.1%] industry) did not reach statistical significance (*P* = .29). However, HAS grading on substantial added clinical benefit reached statistical significance in favor of academia (13 [7.7%] vs 12 [2.6%] in the industry; *P* = .003), whereas Prescrire grading did not (1.8% academia vs 1.3% industry; *P* = .64).

**Conclusions and Relevance:**

More than 70% of medicines that entered the French market during the 10-year period originated in the commercial sector. Although most medicines were not graded as providing clinical benefit, medicines originating in the academic setting were more likely to be graded as conferring clinical benefit than those originating in the commercial setting.

## Key Points

**Question:**

What is the provenance of medicines that entered the French market between 2008 and 2018, and what clinical benefit do they deliver?

**Findings:**

This cross-sectional study found that of 632 medicines in the sample, 464 originated (73%) in the commercial sector, and 168 (27%) in academia or in academic-commercial collaborations. Medicines originating in the academic setting were graded to have better clinical benefit than those originating in the industry.

**Meaning:**

Although the industry invents nearly 3 times more medicines, those originating in academia bring better clinical value.

## Teaser

This cross-sectional study examines the provenance and clinical benefit of medicines that entered the French market between 2008 and 2018 using the Prescrire and Haute Autorité de Santé grading systems.

## Introduction

Policy changes introduced in the 1980s in the US (eg, the Bayh-Dole Act; Public Law 96-517 and the Stevenson–Wydler Technology Innovation Act; Public Law 96-480) and to a lesser extent in Europe provided public sector–supported research institutes with proprietorship and control over their intellectual property.^1,2,3^ Similar to academic organizations, Lincker et al^4^ noted that public-private partnerships (PPP) frequently out-licensed products to larger companies instead of carrying their products through to market authorization. Thus the company that submits an application to the regulatory bodies for approval of a novel therapy may not be the organization that discovered the active pharmaceutical ingredient.^5^

Therefore, the provenance of many medications is not always apparent. This makes it difficult to understand the relevant contributions of academia and industry to the intellectual property underlying newly approved medicines.

From the perspective of patients and clinicians, clinical efficacy is the most important aspect of new therapies. Hence, rigorous comparison of new medicines to standard-of-care therapies are crucial. Different approaches to establishing this additional clinical benefit exist; however, there is no standardized system that is widely used.^6^ For example, clinical benefit grading is used in France and Germany for reimbursement decisions; however, the agreement between these grading systems has been found to be only 50.3%.^7^ The US (Institute for Clinical and Economic Review [ICER])^8^ and Canada^9^ use their own approaches to establishing additional clinical benefit as part of the health technology assessment process. The European Society for Medical Oncology^10^ and American Society of Clinical Oncology clinical benefit grading scales were developed to inform decision-making for new oncology medicines at the patient and population levels, respectively.^11,12^

In France, additional clinical benefit of medicines is assessed by 2 organizations. Haute Autorité de Santé (HAS)^13^ provides a scientific opinion concerning the usefulness, interest in, and appropriate use of new medicines. After a medicine has acquired a marketing authorization, the HAS scientific committee on transparency (Commission de la Transparence) assesses the primary medical benefit (Service Médical Rendu) and the additional medical benefit (Amélioration du Service Médical Rendu [ASMR]) of each new medication. Prescrire,^14^ an independent nonprofit continuing education organization in France, provides health care professionals with clear, comprehensive, and reliable information on added clinical benefit of medicines and diagnostic strategies.

In this study, we establish the provenance of medicines that entered the French market (as a proxy for the European market) between 2008 and 2018. From the existing grading by Prescrire and HAS, we identify the additional clinical benefit of these medicines and determine the degree of concordance between these organizations. We present analyses by therapeutic category, the provenance of medicines, and type of product (combination vs noncombination).

## Methods

### Data Sources

The Prescrire clinical benefit grading of medicines is publicly available information. For this study, a data set (2008-2018) of clinical benefit grading was provided by Prescrire in Excel. Two researchers (P.P. and F.A.) independently reviewed all entries and eliminated vaccines, nutritional supplements, medicines that did not have clinical benefit grading, and duplicate entries. A third researcher (L.O.) quality assured this data preparation process. For medicines that listed multiple indications, the indication with the highest Prescrire-graded clinical benefit during the study period was selected. This decision was based on the fact that the inclusion of medicines designed for multiple indications would result in an inappropriate skew of the frequencies of specific medicine origins. The HAS data (ASMR grading) on clinical benefits were collected from a publicly-available online database.^15^ The data on the US marketing authorization of medicines in this study was sourced from the US Food and Drug Administration (FDA) Approved Drugs Database.^16^

### Identification of Medicines’ Origins

We chose not to limit ourselves to patent information to identify origins of medicines. Although this method was used in previous research studies,^1^ we recognize that the owners of a given patent may not be the original inventors, nor does patent ownership necessarily reflect the underlying basic science discoveries that may have led to a specific patent. Our aim was to identify the first mentioned inventor of the product to establish the origin of a medicinal product. To identify this information, the following sources were considered: pharmaceutical substances (syntheses, patents, and applications^17^); AdisInsight database; the study by Fischer and Ganellin, 2010^18^; previously published literature^1,19,20^ on the origins of specific medicines; the Pharmaceutical Manufacturing Encyclopedia^21^; and Wikipedia. Google Search was used to identify articles, book chapters, and corporate and researcher websites that contain information on the origin of a given medicine.

The medicines’ origins were identified by a group of 5 researchers (A.K., A.S., P.P., F.A., and an independent researcher). The output from each researcher was evaluated and validated by a counterpart. Discrepancies were evaluated by a third researcher (L.O.) who reconciled any discrepancies. Further searches were undertaken to identify additional sources of information as needed.

Medicines’ origins were classified based on the following categories: industry, biotechnology, academia, joint collaborations. The category labeled industry included all medicines originating from the pharmaceutical industry, biotechnology companies, and the pharmaceutical industry in collaboration with biotechnology companies. The category labeled academia included medicines originating in academic institutions alone, academic institutions in collaboration with biotechnology companies and/or pharmaceutical industry.

Combination medicines are those that contain more than 1 active component. Some components of combination medicines had already been identified by the process described above. Origins of other components were identified and categorized using the same methodology.

### Therapeutic Categories

Medicines were assigned to broad therapeutic categories defined by Drugs.com. The 17 therapeutic categories included (1) antiallergenics, (2) anti-infectives, (3) antineoplastics, (4) biologicals, (5) cardiovascular agents, (6) central nervous system agents, (7) coagulation modifiers, (8) gastrointestinal agents, (9) genitourinary tract agents, (10) hormones, (11) immunologic agents, (12) metabolic agents, (13) radiologic agents, (14) psychotherapeutic agents, (15) topical agents, (16) respiratory agents, and (17) miscellaneous. Two independent researchers with pharmacology training performed the initial recoding. Discrepancies were reviewed and resolved by a third independent researcher.

### Clinical Benefit

Prescrire evaluates the therapeutic value of medicines entering the French marketplace on a 7-tiered scale: (1) bravo, (2) a real advance, (3) offers an advantage, (4) possibly helpful, (5) nothing new, (6) not acceptable, or (7) judgment reserved. HAS evaluates the therapeutic value of medicines using a 6-tiered scale: (1) major clinical benefit, (2) important clinical benefit, (3) moderate clinical benefit, (4) minor clinical benefit, (5) nonexistent clinical benefit, or (6) nonapplicable. Because Prescrire and HAS use different grading systems to assign clinical benefit, a simplified system was created that matched the 2 scales, as shown in the Table. Two independent reviewers (P.P. and F.A.) identified the highest level of clinical benefit assigned by HAS for each medicine as well as the corresponding Prescrire grading for the same indication. The resulting matched scale included 3 grades: (1) substantial added benefit, (2) some added benefit, or (3) no added benefit. Notably, medicines without sufficient evidence were marked as *judgment reserved* in the Prescrire scale but were not graded by HAS. For this category, we combined judgment reserved with no added benefit category but present these medicines separately to the aggregate analysis.

**Table.** Clinical Benefit Scales

HAS grading	Prescrire grading	Matched scale
Major	Bravo	Substantial added benefit
Important	A real advance	
Moderate	Offers an advantage	Some added benefit
Minor	Possibly helpful	
Nonexistent	Nothing new	No added benefit
NA	Not acceptable	
	Judgment reserved	

Abbreviations: HAS, Haute Autorité de Santé grading system; NA, not applicable.

### Analysis

We calculated the proportion of frequencies of medicines graded by Prescrire and HAS in the following categories: (1) origin of each medicine, (2) therapeutic category, and (3) clinical benefit. The χ^2^ test was used to analyze the proportions and frequencies of medicines graded by Prescrire and HAS in the previously specified categories. Following the calculation of proportions and frequencies for each category, the results were converted to the matched scale (Table). Subsequently, the number of medicines with identical Prescrire and HAS clinical benefit gradings was calculated to establish the degree of concordance using the κ test. This test quantifies the level of agreement between 2 grading systems, considering the possibility of agreement due to chance alone (κ = 0) and the highest level of agreement (κ = 1). All statistical analyses were performed using STATA statistical software (version 16; STATA Corp) with significance levels set at *P* < .05. We also calculated percentage of medicines that obtained marketing authorization in the US.

## Results

Of the 1177 medicines in the original Prescrire database, 632 remained for analysis after we eliminated vaccines (n = 16), nutritional supplements (n = 36), duplicates (ie, medicines for multiple indications; n = 341 [note that the indication with the highest clinical benefit available was kept on the list for analysis]), and those for which no judgment was listed in either Prescrire and HAS (n = 152).

Of these 632 medicines, 529 were identified as single medicines and 103 as combination medicines. Most of these medicines (464 [73.4%]) originated in the commercial setting, and slightly more than a quarter (168 [26.6%]) were invented in the academic setting. When assessed by subcategory, more than half of the medicines originated in the pharmaceutical industry (350 [55.4%]), followed by academic institutions (112 [17.7%]) and biotechnology companies (101 [16.0%]). Sixty-nine (10.9%) products were a result of joint collaborations. The most prevalent therapeutic categories were antineoplastics (114 [18.0%]), followed by anti-infectives (97 [15.3%]), central nervous system agents (71 [11.2%]), and metabolic agents (62 [9.8%]). Overall, 555 medicines (88%) received marketing authorization in the US.

### Clinical Benefit

**Figure 1.**
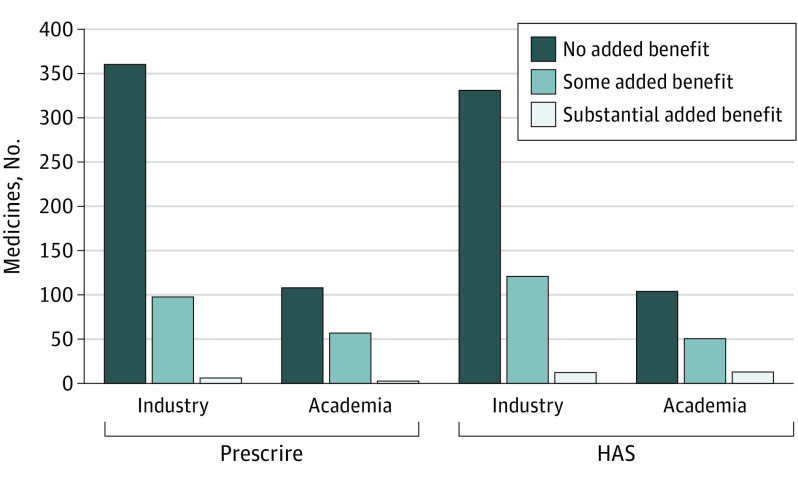
Clinical Benefit Grading of 632 Medicines by Prescrire and Haute Autorité de Santé (HAS)

As shown in Figure 1, Prescrire graded 360 (77.6%) industry originated and 108 (64.3%) academia originated medicines to have no added clinical benefit (*P* = .001). HAS assigned such grading to 331 medicines (71.3%) that originated in the industry vs 104 (61.9%) that originated in the academic setting (*P* = .02). Based on Prescrire grading, academia invented more medicines delivering some added benefit: 57 (33.9%) vs 98 (21.1%) originated by industry (*P* = .001). For HAS grading on some added benefit, 51 medicines (30.4%) originating in academia vs 121 medicines (26.1%) originating in industry, did not reach statistical significance (*P* = .29). However, Prescrire grading on substantial added clinical benefit did not reach statistical significance in favor of academia, with 3 medicines (1.8%) originating in academia vs 6 medicines (1.3%) in industry (*P* = .64), whereas HAS grading did, with 13 medicines (7.7%) originating in academia vs 12 medicines (2.6%) in the industry (*P* = .003).

### Concordance of Clinical Benefit Grading Between Prescrire and HAS

The actual agreement between the Prescrire and HAS grading systems was 66.9%, compared with the expected agreement of 57.7%. This resulted in a κ statistic of 0.22 (95% CI, 0.15-0.29), which represents poor agreement.^22^

### Clinical Benefit by Therapeutic Category

**Figure 2.**
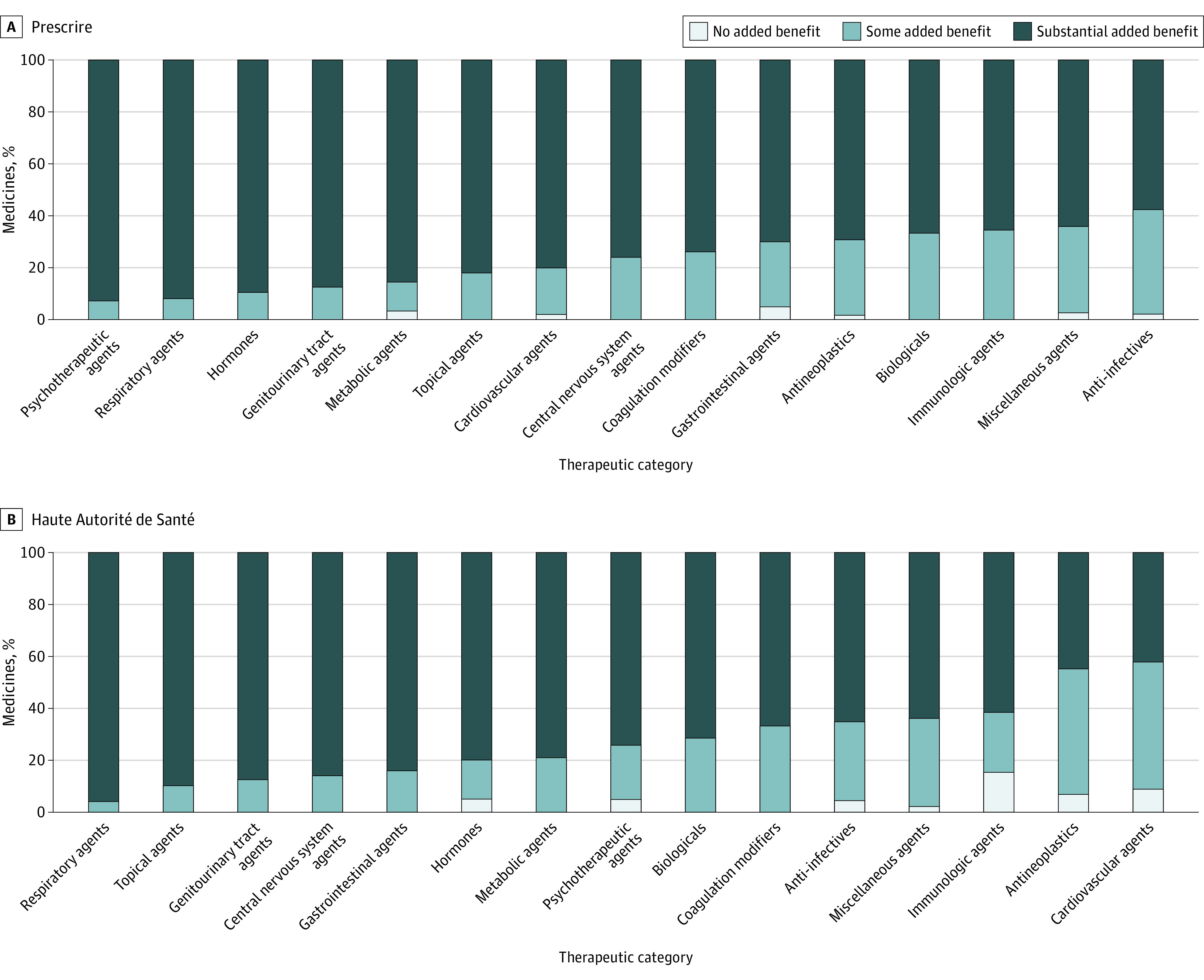
Clinical Benefit Grading Therapeutic Category A, Prescrire clinical benefit grading. B, Haute Autorité de Santé clinical benefit grading.

Results from the Prescrire grading system revealed that the therapeutic categories with the highest percentage of medicines that provided no added benefit were psychotherapeutic agents (13/14 [92.9%]), respiratory agents (23/25 [92.0%]), and hormones (34/38 [89.5%]; Figure 2, A and B). By contrast, HAS identified respiratory agents (24/25 [96.0%]), topical agents (35/39 [89.7%]), and genitourinary agents (7/8 [87.5%]) as the therapeutic categories with the highest percentages of medicines that provide no added benefit. Antineoplastics represent the largest group of medicines in our sample size, at 114 (18%). HAS graded antineoplastics as the medicines with the highest added clinical benefit: 48 (42%) had some added benefit and 13 (9%) offered substantial added benefit, whereas Prescrire assigned such grading to 33 (29%) and 2 (2%) antineoplastics medicines, respectively.

### Combination vs Noncombination Medicines

Of the 632 medicines identified in this study, 529 (83.7%) were noncombination (also known as single) medicines and 103 (16.3%) were combination medicines. Our findings revealed that 255 of the single medicines (70.0%) and 95 of the combination medicines (92.0%) originated from industry. The 3 most common therapeutic categories that included single-medicine formulations were antineoplastics (111 [21.0%]), anti-infectives (75 [14.1%]), and central nervous system agents (60 [11.3%]). By contrast, the 3 most common therapeutic categories that included combination medicines were anti-infectives (22 [21.4%]), topical agents (16 [15.5%]), and metabolic agents (15 [14.5%]). The full set of results are available in an online data repository.^23^

### Clinical Benefits of Combination Medicines

**Figure 3.**
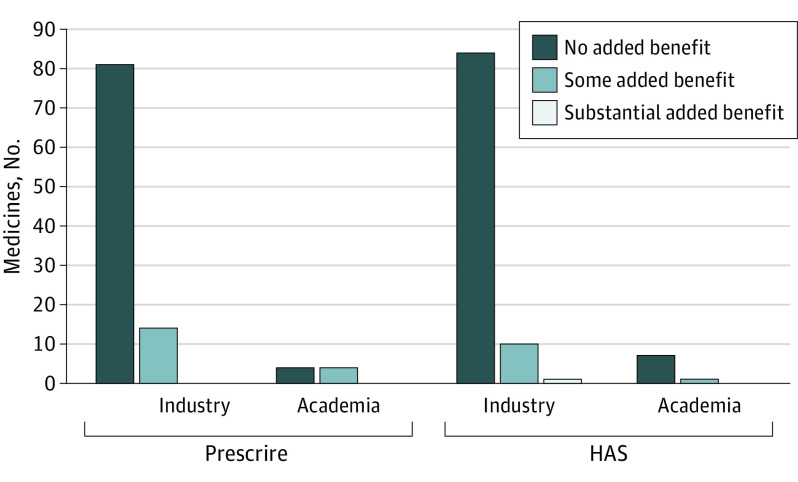
Clinical Benefit Grading of 103 Combination Medicines HAS indicates Haute Autorité de Santé.

Prescrire graded 81 (85%) combination medicines originating in the industry and 4 (50%) originating in academia as having no added benefit. HAS graded 88% of combination medicines, regardless of origin, as no added benefit (84 for industry medicines and 7 for academia). Only 1 combination product that originated in the industry was graded by HAS as a substantial added benefit, and none received this grading from Prescrire (Figure 3).

## Discussion

This analysis of 632 medications introduced to the French market between 2008 and 2018 found that approximately 1 in 4 drugs originated in the academic setting. We also found that medicines originating in academia were more likely to be characterized as providing added clinical benefit. However, regardless of the origin of the medicines, more than two-thirds of new drugs provided no additional clinical benefit compared with those that were already available. Combination products predominantly come from industry, and an even greater proportion of these products (88% according to HAS) compared with single medicines are characterized as having no added benefit.

These findings are likely generalizable beyond France. Most medicines in our sample had first obtained regulatory approval from the European Medicines Agency (EMA) and are available throughout Europe and the US. These results are also consistent with previously established trends. Between 1981 and 2000, Prescrire graded only 74 (3%) of the nearly 2300 new medicines or new indications for existing medicines as representing major or important therapeutic gains.^24^ The Institute for Quality and Efficiency in Health Care (IQWiG) in Germany also highlighted a decline in the development of innovative drugs accompanied by an increase in the number of drugs with no added clinical benefit.^25^

Multiple processes are available for establishing additional clinical benefits of medicinal products^8,9,10,11,26^; however, the use of these scales is inconsistent, and results from these grading systems may not be comparable. It would make sense to standardize and harmonize this process at the international level. For example, we found that antineoplastics were graded by HAS as medicines with the highest additional clinical benefit, whereas Prescrire graded 4 other therapeutic areas higher than antineoplastics.

The discovery and development of new therapies has and will likely continue to require contributions from academic institutions and the biopharmaceutical industry.^27^ The systematic review by Kesselheim and colleagues^26^ concluded that therapeutic value measures hold the greatest promise for evaluating the effectiveness of investments in drug development by public and private sources and should continue to be central to assessments of the output of the pharmaceutical industry, whether one is measuring innovation, productivity, value creation, or other markers. However, based on these findings as well as previous research^24,25^ many products on the market offer low or no added clinical benefit. This means that regulatory authorities consistently grant authorizations to products with marginal or nonexistent clinical effectiveness and prescribers offer them to patients. Information available to clinicians and patients does not clearly highlight the added value of new medicines. To promote real clinical innovation, incentives should specifically reward the development of drugs with proven added value for patients.^28^

Finally, we found that establishing provenance of medicinal products is a laborious process. Data on the origin of many medicinal products are not easy to find or verify if sources report diverging information. The field would benefit from a pharmacological repository that lists and verifies provenance of medicinal products. The data we collated in this study^23^ can serve as an important addition to the creation of such a resource.

### Limitations

This study has several limitations. For each medicinal product that had more than 1 indication, we only considered it once in the indication of the highest clinical benefit grading available from Prescrire. This permitted us to provide a best-case scenario of benefit judgment without skewing data on origin frequencies.

We recognize that judging clinical benefit is a bias-prone process and is subject to changing evidence. We found that comparing grading from the HAS and Prescrire clinical benefits scales is not straightforward. We created a simplified matched scale (Table) but still found poor agreement between rating systems. Of note, 17% of medicines graded by Prescrire as judgment reserved were assigned to the no added benefit category which, with further evidence availability, might have moved to a different benefit category. Regardless, most drugs were rated as not conferring substantive benefit in either system.

Although we performed a detailed search for the origins of all the medicines, it was not always possible to identify or confirm the original source of data. Of note, a recent study by Kinch and colleagues^29^ supports our finding—their analysis of all medicines approved from 2001 through 2019 in the US determined that academic inventors contributed to more than a quarter of these medicines. Our data set^23^ that references the origins of specific medicines included in this study is available in the public open-source repository on Zenodo and can be rechecked and corrected should more accurate information be identified by other researchers.

## Conclusions

This cross-sectional study established that three-fourths of the new medicines entering the French market between 2008 and 2018 delivered no additional clinical benefit regardless of the therapeutic category, provenance, or the type of the product (combination vs single-medicine formulation). More than 70% of medicines that entered the French market during the 10-year period originated in the commercial sector. In our sample, medicines originating in the academic setting were graded to have better clinical benefit than those originating in the commercial setting.

## Notes

Supplement. Data Sharing Statement
