# Supplementary material for: Provenance and Clinical Benefit of Medicines Introduced to the French Market, 2008 to 2018
Source: JAMA Intern Med. 2023 Nov 20;184(1):46–52. doi: 10.1001/jamainternmed.2023.6249 (PMC10660249; doi:10.1001/jamainternmed.2023.6249)
Supplement: Supplement. — Data Sharing Statement [file jamainternmed-e236249-s001.pdf]

Data Sharing Statement

Osipenko. Provenance of Medicines and Their Clinical Benefit—A Review of Drugs Introduced to the French Market (2008-2018). *JAMA Intern Med*. Published November 20, 2023. doi:10.1001/jamainternmed.2023.6249

Data
Data available: Yes
Data types: Data (not involving human participants)
How to access data: Provenance, Added Clinical Benefit and Therapeutic Area of 632 medicines that entered the French market between 2008 and 2018. Data set Available online https://zenodo.org/record/8169147
When available: beginning date: 04-13-2023

Supporting Documents
Document types: None

Additional Information
Who can access the data: n/a
Types of analyses: n/a
Mechanisms of data availability: n/a
Any additional restrictions: n/a
